# How efficient are facial masks against COVID-19? Evaluating the mask use of various communities one year into the pandemic

**DOI:** 10.3906/sag-2106-190

**Published:** 2021-12-17

**Authors:** Aslıhan CANDEVİR, Cem ÜNGÖR, Figen ÇİZMECİ ŞENEL, Yeşim TAŞOVA

**Affiliations:** 1 Department of Infectious Diseases and Clinical Microbiology, Faculty of Medicine, Çukurova University, Adana Turkey; 2 Department of Oral and Maxillofacial Surgery, Faculty of Dentistry, Karadeniz Technical University Trabzon; 3 Health Institutes of Turkey, Ankara Turkey

**Keywords:** COVID-19, efficacy, face mask, pandemic, SARS-CoV-2

## Abstract

Face masks are devices worn over the mouth and nose to protect against splashes, infectious respiratory droplets, or aerosols generated during breathing or coughing according to their filtering capacity. Medical masks, respirators, or cloth masks have been used for source control and for the protection of the exposed. After the first case on March 11, 2020, in Turkey, National COVID-19 Scientific Advisory Board published various contents for the correct use of masks. Medical face masks have been used in healthcare settings for both source control and potential personal protection before the COVID-19 pandemic. Adverse events associated with using masks are very sparse and mainly associated with tight-fitting respirators or dermatitis due to prolonged use and should not be a reason for refusal to use. Studies suggest the use of masks mainly in the healthcare facilities but also in the community for source control of people who have respiratory symptoms of communicable diseases other than COVID-19. They are likely to be acceptable if recommended, particularly in more severe epidemics and pandemics. Metaanalysis, case control, cross sectional, cohort, retrospective, retrospective cross sectional, research, randomized controlled, and controlled comparison studies were reviewed on the protective effect of masks on COVID-19 with laboratory evidence. Optimum use of face masks with additional precautions has been found to be useful controlling the spread of the respiratory viruses such as SARS-CoV-2 in most of the studies and metaanalyses. As a conclusion, the recent evidence in COVID-19 pandemic is consistent with the previous studies which have shown association between face mask use and decreased risk of viral infections, and medical face mask use should be encouraged both for the community and healthcare facilities along with other infection control measures such as hand hygiene, during outbreaks when there is widespread community transmission.

## 1. Introduction

### 1.1. Properties of face masks

**Figure 1:**
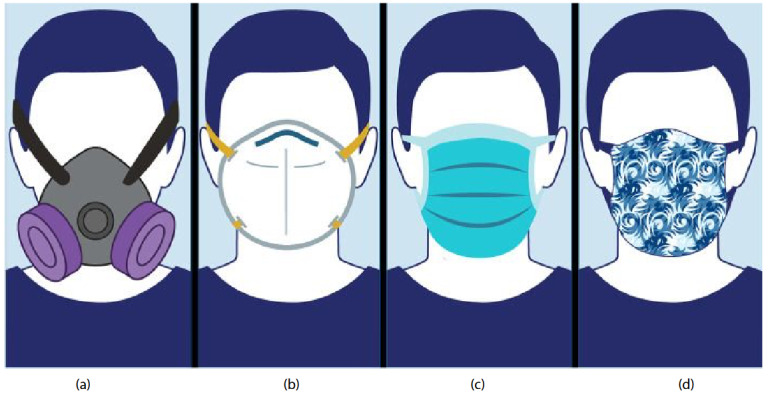
Types of face masks. (a) Elastomeric respirators, tight fitted, half facepiece or full facepiece respirators made of synthetic or natural rubber material, reusable. (b) An N95 filtering facepiece respirator is designed to achieve a very close facial fit and very efficient filtration for airborne particles. (c) A surgical mask/medical face mask is a personal protective equipment worn by health professionals during medical procedures. (d) A cloth mask, or a cloth face covering, which is not standardized or regulated

Face mask is a term used for a nonmedical/medical face mask or a respirator which is worn over the mouth and nose to prevent the splash and inhalation or release of infectious respiratory droplets, or for harmful substances. Types of face masks were demonstrated in Figure 1a as elastomeric respirators, in Figure 1b as N95 filtering facepiece, in Figure 1c as surgical mask, and in Figure 1d as cloth mask. Infectious respiratory droplets can be generated by breathing, speaking, coughing, or sneezing [1]. Masks can be used for source control or for the protection of the wearer. Source control is when it is used to prevent the spread of infectious respiratory particles like droplets or aerosols, and it is for the protection of others in the environment. Nonmedical face masks (e.g., cloth masks) used in community include many self-made forms or commercial masks, including masks made of disposable materials or textile which can be washed and reused. Because of not being standardized, they are not compatible for the use in healthcare facilities by healthcare workers (HCWs). The main three categories of masks are summarized in Table 1. 

**Table 1:** Summary of three main mask categories.

Masks	Characteristics	Advantages	Disadvantages
Respirators(e.g., N95 masks)	· Protects from aerosols/droplets· Made of 4 layers, usually polypropylene and other materials· Requires certification by authorities, NIOSH in the US or equivalent organizations in other countries · EN 149: 2001 +A1:2009, TSE EN 149 standards for European Union and Turkey retrospectively	· Designed to be tightly fitted and has a tight seal· High filtration efficiency· Contains electrets to filter particles electrostatically· Recommended for healthcare workers performing aerosol generating procedures · May be oil resistant depending on model· May be fluid resistant depending on model, e.g., surgical N95	· Expensive· Not readily available· Designed for single use, or when possible, complexity of decontamination and reuse techniques
Surgical masks	· Prevents aerosol/droplet spread instead of protecting the wearer· Usually made up of 3 layers of melt-blown polypropylene · Approved by FDA, EN 14683, EN 14683+AC standards, but has a wide variety of masks	· Cheap· May use electrets· Flame and fluid resistant · No significant differences for H1N1 infection rate for healthcare workers wearing either N95 or surgical masks	· Loose fitted· Single use· Not suitable for high-risk environments and aerosol generating procedures
Clothmasks	· Made up of various fabrics (cotton,silk, nylon, etc.)· Not regulated by any agency	· Cheap and easy to produce· Widely available for public· Can be washed and reused, · May use electrets, depending on material used	· No standardization in design and material· Poor filtration efficiency and may decrease by washing· Not fitted· Not recommended for HCWS

Medical masks or surgical masks are loose-fitting disposable medical devices which can protect the users from large respiratory droplets or splashes produced by sneezing or coughing as physical barrier but not aerosols and airborne infection [2,3]. They vary in thickness and permeability. A medical face mask can also be used as source control to stop the spread of large respiratory droplets from the person wearing them [4]. Requirements for medical face masks, including the duration of use, are defined in the European Committee for Standardization’s published standards. The EN 14683 standard, a European standard, describes the requirements and test methods for medical face masks. This standard has also been published in our country by the Turkish Standards Institute (TSE) with the following title: TS EN 14683+AC Medical face masks - Requirements and test methods. 

Respirators are maintenance-free, tight-fitting disposable personal protective equipment which are classified according to European Union (EU) standard EN 149: 2001 +A1:2009 or NIOSH USA standards. They are designed to protect the wearer from airborne microorganism exposure according to their types and filtering degree. Filtering performance strongly depends on fitting and different devices should be tested by HCWs to find the best fitting size and model for their face. The protection degrees of respirators are determined by their filtering capacity of 0.3 µm and larger particles. According to EU and US standards, the degrees of protection are as follows; FFP1: 80%, FFP2: 94%, FFP3: 99%, and N95: 95%, N99: 99%, N100: 99.9%, FFP for EU and N for US. An N95/N99 respirator is the United States is equivalent of FFP2/FFP3 respirators in EU as defined by U.S. standard NIOSH 42 CFR, part 84. The standard in question has been published in our country by the Turkish Standards Institute (TSE) with the following title: TS EN 149 Respiratory protective devices - Half masks with filters for protection against particles - Features, tests and marking.

### 1.2. Potential adverse effects of face mask use

Wearing a face mask may cause anxiety and difficulty in breathing and this may be seen more in people with underlying respiratory or psychiatric disease. On the other hand, it is not proven by scientific evidence that wearing a face mask aggravates respiratory or other diseases [5,6]. Several studies did not find significant physiological effects on people wearing a face mask even during intense exercise [7–10]. However, there are a lot of cases reporting adverse skin reactions, such as erythema and pruritus particularly due to the extended use [11–14]. It is also important that the tight-fitted face masks like respirators generally results in problems of tolerability, discomfort, and headaches [13]. Communication breakdown is an additional problem that masks may cause, especially among people with hearing impairment, particularly because the missing speech reading cues [16,17].

The availability of medical face masks may be limited sometimes during the pandemic. This can cause high costs or inappropriate reuse of masks which could result in an increased risk of self-contamination or individuals’ incompliance with the policies [18]. 

It should not be forgotten that the production and disposal of large amounts of face masks made of synthetic materials due to universal mask use may have a detrimental effect on the environment if not appropriately managed [19].

The impact of using face masks should depend on the prevalence of COVID-19 in the community. In places without significant community transmission of COVID-19, the potential harms and costs may outweigh the benefits [19,20]. In implementing policies on the widespread use of face masks for the prevention of COVID-19 in the community, these potential barriers and adverse effects should also be considered [21]. 

### 1.3. Mask use in Turkey

The first detected case of COVID-19 in Turkey of the COVID-19 epidemic, which spread worldwide, was announced by the Ministry of Health on March 11, and the first death due to the virus occurred on March 15, 2020. At the beginning of April, in addition to the curfew and other measures for certain age groups, masks were made mandatory in public areas such as markets, and it was decided to distribute masks free of charge. In this period, brochures and informative films about the use of medical and cloth masks in the society were prepared by the National COVID-19 Scientific Advisory Board for the correct use of masks. Similarly, detailed instructions and brochures on the use of medical masks and respiratory masks in health institutions were prepared. Today, compulsory use of masks in community continues along with other measures.

## 2. Use of face masks in preventing influenza, SARS, and other respiratory viral infections

Medical face masks have been used in healthcare settings for both source control and potential personal protection before the COVID-19 pandemic. They were recommended mainly in the healthcare facilities but also in the community for source control of people who have respiratory symptoms of communicable diseases. They are likely to be acceptable if recommended, particularly in more severe epidemics and pandemics. However, face masks may not be appropriate in some conditions such as during sleep and it should not be forgotten that compliance may be lower in some areas and particular groups.

The main uses of medical face masks were for preventing the transmission of tuberculosis and influenza as means of source control. The evidence of face masks in preventing infectious diseases other than SARS-CoV-2 in the community and healthcare facilities are summarized below [22,23]. 

### 2.1. Community

In a metaanalysis on efficacy of the use of face masks in community by WHO, ten randomized controlled trials (RCTs) were reviewed. Most of the trials investigated the combined effect of face masks with improved hand hygiene. In the pooled analysis, a 22% relative risk reduction was found in laboratory-confirmed influenza (RR: 0.78, 95% CI: 0.51–1.20, I2 = 30%, p = 0.25) in the face mask group, and 8% reduction of in the face mask group regardless of hand hygiene (RR: 0.92, 95% CI: 0.75–1.12, I2 = 30%, p = 0.40), but the evidence was insignificant. Low compliance in mask use was suggested to be reducing their effectiveness. As a conclusion, they recommended face masks to be worn by asymptomatic people in the community in severe epidemics or pandemics due the potential effectiveness of this measure, although there was no evidence of them reducing transmission. They concluded that there were important knowledge gaps in the mechanisms of person-to-person transmission of influenza and high-quality RCTs investigating the efficacy of face masks against laboratory confirmed influenza were needed [23].

Eight RCTs showed inconsistent and statistically insignificant results. In two of the RCTs, a statistically significant favorable effect was found in the subgroup that included use of a medical face mask within 36 h from symptom onset. In most of these RCTs, medical face masks were used both by ill in means of source control and the healthy susceptible people. It is difficult to distinguish if the effect is related to personal protection or from source control [24–28]. There was heterogenicity between studies and deviations from interventions in several studies such as compliance problems in the intervention groups whereas several participants in the control groups were using medical face masks in some studies [29,30].

Two case-control studies investigated the transmission of SARS in the community and statistically significant effect was detected in favor of the use of face masks. (OR 0.3–0.36). A cross-sectional study also demonstrated a favorable but not statistically significant effect [31–33]. 

Another recent metaanalysis including RCTs and case-control studies suggests enough evidence for medical masks’ effectiveness in community to prevent transmission of respiratory viral infections. This metaanalysis showed a significant effect of medical mask use in preventing the transmission of respiratory infections, including SARS-CoV-2 (OR = 0.66) with or without other interventions. Subgroup analysis which includes only RCTs also demonstrated significant protection of medical facemask in preventing influenza and influenza-like illness (ILI) (OR = 0.71 and OR = 0.70, respectively) [34]. 

Medical mask use in community should be strongly encouraged in outbreaks, particularly when there is widespread community transmission and where physical distancing could not be provided. However, face masks should not be a replacement for other public health measures such as physical distancing or hand hygiene. 

### 2.2. Healthcare settings

According to a metaanalysis by Offeddu et al. [2] which included 6 RCTs and 23 observational studies, in RCTs masks and respirators provides statistically significant protection against ILI (RR = 0.34) and clinical respiratory illness (CRI) (RR = 0.59). Only two RCTs investigated the respiratory infection risk in healthcare workers (HCWs) wearing personal protective equipment (PPE) continuously and not wearing PPE for control group [35,36]. The metaanalysis suggested a protective effect against viral infections which were laboratory-confirmed but it was insignificant. N95 respirators demonstrated greater and statistically significant protection against CRI (RR = 0.47) and laboratory-confirmed bacterial infection (RR = 0.46), but no superiority for viral infections or ILI when compared to masks [2]. 

Protective effect for masks against severe acute respiratory syndrome (SARS) was reported by most of the case-control studies. Four out of the five case-control studies demonstrated a favorable statistically significant effect (adjusted OR range: 0.08–0.29) [32,37–40]. Cohort studies reported less pneumonia and moderate protection against SARS-CoV infection which was laboratory confirmed. Metaanalysis of these observational studies provided evidence of significant protective effect against SARS (OR = 0.13 for masks and OR = 0.12 for respirators). There was no significant difference between N95 respirators and medical masks in means of protection of HCWs from SARS [2]. 

Early in the outbreak of H1N1 pandemic in 2009, the effect of masks and respirators was investigated in HCWs. Pandemic H1N1 seroconversion was 21% (9/43) in HCWs treating H1N1 patients without respiratory PPE and 0 % in others [41]. Similarly, in a cohort study from Hong Kong, HCWs using medical masks during whole patient contacts remained healthy. In two matched case-control and two cross sectional studies, no association was found between compliance with respiratory PPE use and pH1N1 infection [2]. 

A recent RCT investigating difference of the preventive effect between N95 and medical masks, among outpatient health care personnel, revealed no significant difference in the incidence of laboratory-confirmed influenza [42]. 

There is evidence supporting medical mask use in medical facilities as part of the infection control programs to mitigate the risk of CRI and ILI among HCWs and N95 respirators may have greater protection. Medical masks and respirators are also found effective for SARS but not for H1N1. 

## 3. SARS-CoV-2 and masks

A total of 12 studies, including metaanalysis, case control, cross sectional, cohort, retrospective, retrospective cross sectional, research, randomized controlled, and controlled comparison studies, conducted in China, United Kingdom, USA, Thailand, Uganda, Ethiopia, South Korea, Canada, and Iran were reviewed on the protective effect of masks on COVID-19 [43–49]. All patients had laboratory evidence of SARS-CoV-2. The researchers investigated healthcare workers, nonprofessional populations, and contacted people in these articles. Most of the studies revealed that optimum use of face masks with additional precautions controlled the spread of the respiratory viruses such as SARS-CoV-2. Most of the studies were retrospective and demonstrated in favor of using mask in healthcare settings, but only RCT failed to show efficacy. The summary of the studies is demonstrated in Table 2.

**Table 2:** The summary of studies on the effectiveness of face masks for SARS-CoV-2 infection.

Study	Country	Population	Study design	The type of mask	Other preventive applications	Results
Wang et al.2020	China	Healthy workers (doctors and nurses)	Retrospective	N95	Disinfection and hand washing	Reduction in the infectious risk of 2019-nCoV in doctors and nurses
Bundgaard et al.2020	Denmark	Adults outdoor more than 3 h per day without occupational mask use	Randomized controlled trial	Surgicalmask	-	Surgical masks in addition with other public measures did not reduce the SARS-CoV-2 infection rate
Ma et al.2020	China	Experimental	Researcharticle	N95, surgical mask, cotton mask	Hand hygiene	Wearing effective masks and hand hygiene, may slow therapid spread of the virus
Fan et al.2020	China	Chinese citizensliving in Iranand subsequently evacuated	Cohort	Surgicalmask	Travel restrictions	Restricting gatherings and wearing facemasks can decrease transmission of COVID-19
Doung-ngern et al.2020	Thailand	Asymptomatic contacts ofCOVID-19 patients	Case control	Surgicalmask	Handwashingand social distancing	Wearing masks, handwashing, and social distancing in public is suggested to protect against COVID-19
Mboowa et al.2021	Uganda	High risk individuals (polices, market workers, healthcare workers)	Retrospective Cross sectional	Face mask	Health education programs	Face masks in healthcare and community settings prevent the transmission of COVID-19
Natnael et al.2021	Ethiopia	Taxi drivers	Cross sectional	N95, surgical mask, cotton mask	-	Prevalence of mask wearing among taxi drivers was low. The significantly associated factors were determined

The metaanalysis by Chu DK et al. investigating use of surgical masks among other measures supports physical distancing of 1 m or more. Face mask use was found to be related with a great reduction of infection risk (n = 2647; aOR: 0.15, 95% CI: 0.07 to 0.34). Stronger associations were found with respirators compared with surgical/medical masks. Eye protection was also found to be associated with less risk of infection [50]. 

Another metaanalysis of 21 studies suggests that mask use provides a statistically significant protective effect (OR = 0.35). Mask use by HCWs and public can decrease the risk of respiratory virus infection by 80 % (95% CI = 0.11–0.37) and 47 % (95% CI = 0.36–0.79). Protective effect of mask wearing was higher in particular regions of the world than others (in Asia OR = 0.31, in the Western countries OR = 0.45). Masks had a protective effect against SARS-CoV-2 (OR = 0.04) along with other respiratory viruses; influenza (OR = 0.55), SARS (OR = 0.26). They concluded that masks can be helpful as an adjunctive method in the COVID-19 outbreak [51]. 

Fourteen studies were included in the metaanalysis from the UK. While observational and preclinical studies finding significant benefit of mask use in preventing SARS-CoV-2 transmission RCTs failed to demonstrate this effect. Eleven of RCTs studying other respiratory diseases than SARS-CoV-2 found no significant benefits for either ILI or viruses which were laboratory confirmed. Only one RCT found a significant benefit of surgical masks over cloth masks. They emphasized the need for evidence from RCTs which reports outcomes such as compliance and comfort [52].

Four articles of a total of 7688 participants were included in a recent metaanalysis. The result has shown a significant decrease in infection with face mask use (the pooled RR = 0.12 95 %CI [0.06, 0.27] P < 0.001). The main limitation of this metaanalysis is that studies included had nonrandomized designs [53].

Eventually, a metaanalysis comparing N95 and medical mask use among HCWs showed that continuous wearing of N95 respirators may have the best protection against viral respiratory diseases. According to researchers, surgical masks needed to be replaced frequently for better efficacy. They concluded more RCTs during this COVID-19 epidemic were needed for further analysis.

## 4. Conclusion

As a conclusion, the recent evidence in COVID-19 pandemic is consistent with the previous studies which have shown association between face mask use and decreased risk of viral infections, and medical face mask use should be encouraged both for the community and healthcare facilities along with other infection control measures such as hand hygiene, in the course of outbreaks when there is widespread community transmission. 

## Informed consent

No ethical review board approval or informed consent is needed.
